# Similar Shift Patterns in Gut Bacterial and Fungal Communities Across the Life Stages of *Bactrocera minax* Larvae From Two Field Populations

**DOI:** 10.3389/fmicb.2019.02262

**Published:** 2019-10-09

**Authors:** Zhichao Yao, Qiongke Ma, Zhaohui Cai, Muhammad Fahim Raza, Shuai Bai, Yichen Wang, Ping Zhang, Haiquan Ma, Hongyu Zhang

**Affiliations:** State Key Laboratory of Agricultural Microbiology, Institute of Urban and Horticultural Entomology, College of Plant Science and Technology, Huazhong Agricultural University, Wuhan, China

**Keywords:** *Bactrocera minax*, bacteria, fungi, development, dynamic change

## Abstract

*Bactrocera minax* (Enderlein) (Diptera: Tephritidae) is an oligophagous insect pest that damages citrus fruit, especially in China. Due to larvae living within a highly septic environment, a wide variety of microorganisms exist in the larval gut of *B. minax*. However, a systematic study of the intestinal microbiota of this harmful insect pest is still lacking. Here, we comprehensively investigated the larval gut microbiota of *B. minax* in two field populations from Zigui (developed in orange) and Danjiangkou (developed in mandarin orange). We observed a dominance of Proteobacteria and Firmicutes in these bacterial communities, and Enterobacteriaceae was the predominant family throughout the larval stage. However, most of the identified fungal sequences were annotated as being from either Ascomycota or Basidiomycota phyla. Although there was a difference in the structure of the microbial communities between the two populations, the dynamic change patterns of most of the members of the microbiota were similar across the lifespan of larvae in both populations. The relative abundances of the Acetobacteraceae, Leuconostocaceae, and Lactobacillaceae gut bacteria as well as the Pichiaceae, Sebacinaceae, and Amanitaceae fungi increased throughout development, and these microorganisms stably resided in the larval gut. Furthermore, the dynamic changes of the functions of gut bacterial communities were inferred, and there was a significant increase in carbohydrate metabolism across the lifespan of larvae in both groups. Spearman correlation analysis showed that Acetobacteraceae, Lactobacillaceae, and Leuconostocaceae displayed a positive correlation with fructose and mannose metabolism, an important pathway of carbohydrate metabolism, highlighting the potential roles of these prevalent microbial communities in host biology.

## Introduction

The insect gut is an ecosystem that is accompanied by complicated interactions with microbes: numerous studies have revealed the diverse compositions and structures of the microbial communities in the insect gut (Engel and Moran, 2013; Douglas, 2015), ranging from several hundred species in termites (Hongoh et al., 2005; Brune and Dietrich, 2015), to less than a hundred species in lepidopterans (Broderick et al., 2004; Robinson et al., 2010; Pinto-Tomás et al., 2011; Paniagua et al., 2018), and to almost a complete absence in aphids (Grenier et al., 2006). Gut microbes influence host-associated biology and ecology in diverse ways, including by degrading of recalcitrant food, providing of specific nutrients (Engel and Moran, 2013), regulating energy balance (Yamada et al., 2015), and promoting host growth and development (Shin et al., 2011). Additionally, some studies have also revealed that microbiota play roles in detoxification (Ceja-Navarro et al., 2015) and in protection from predators, parasites, and pathogens (Endt et al., 2010; Stecher and Hardt, 2011), thereby influencing mating (Sharon et al., 2010), maturation and development of the immune system (Weiss et al., 2011). Changes in the intestinal microbiota composition caused by antibiotics, a dysregulated immune system or irradiation reduce the host’s ecological fitness (Ryu et al., 2008; Raymann et al., 2017; Cai et al., 2018). Indeed, the insect gut microbiota is considered an essential component of host insects, and maintenance of a healthy composition of the gut microbiota is important for host life.

*Bactrocera minax* is an oligophagous and univoltine species that damages citrus fruit, mainly in southern/central China, and many Southeast Asian countries (Wang and Luo, 1995; Drew et al., 2006). Adult flies live by feeding on substances such as honeydew and reach sexual maturity approximately 3 weeks after emerging. Females prefer laying eggs in young fruits and oviposit from 50 to more than 200 eggs (Xia et al., 2018). Interestingly, the eggs last for approximating 1 month before hatching, and the entire lifespan of the larvae is completed in the fruit, which in turn, causes fruits to brown and rot, leading to premature drop. And *B. minax* experience a diapause when overwinter in the soil as pupae (Chen et al., 2016). To control this agricultural pest, a number of studies are currently being performed to understand the biology and ecology of *B. minax* (Xiong et al., 2016; Wang et al., 2018). However, there is still a lack of research on the bacterial and fungal compositions of the larval gut of *B. minax*, as well as on the shift patterns and functions of the core microbiota.

In the present study, we investigated the dynamic changes of bacterial and fungal communities in the gut of *B. minax* larvae at different life stages from two field populations. Apparently, the microbial communities shifted across the lifespan of the larvae in correlation with host developmental changes. Most bacteria and fungi present in the gut displayed a similar pattern of prevalence across the different larval stages of *B. minax*. In addition, we inferred changes in metabolic pathways during the development of larvae, which were influenced by the shifts in bacterial communities.

## Materials and Methods

### *B. minax* Larval Sample Collection

Maggot-infested fruits were collected from Zigui and Danjiangkou in Hubei Province. Before dissection, maggot fruits were surface sterilized by immersion in 70% ethanol for 3 min and rinsed three times in sterile phosphate-buffered saline to avoid contamination from the environment. The classification standard for different instars was as follows: (1) first instar larvae, body length of less than 5 mm and a milky white color; (2) second instar larvae, body length of 5–14 mm and a creamy yellow color; and (3) third instar larvae, body length of 13–18 mm and a maize-yellow color. After collecting different development stages, we dissected the whole gut from the larvae. Before dissection, the larvae were surface sterilized by immersion in 70% ethanol for 3 min and rinsed three times in sterile phosphate-buffered saline. Four independent cohorts of larvae were dissected and used as biological replicates. Tissue samples were homogenized in an automatic sample Precellys-24 homogenizer (Shanghai Jingxin Industrial Development Co., Ltd., Shanghai, China) at 70 Hz/s for 60 s with a 10 s interval.

### DNA Extraction and High-Throughput Sequencing

DNA was isolated from 30 larval guts per replicate using the E.Z.N.A^TM^ Soil DNA kit (Omega, Norcross, GA, United States) following the manufacturer’s instructions. To confirm successful DNA isolation, a NanoDrop ND-1000 spectrophotometer (Thermo Fisher Scientific, Waltham, MA, United States) and agarose gel electrophoresis were used to check the quantity and quality of extracted DNA, respectively. The 16S ribosomal RNA (rRNA) gene-spanning variable regions (V3–V4) and fungal internal transcribed spacer (ITS1) region were amplified using the following primer pairs: 338F/806R (5′-ACTCCTACGGGAGGCAGCA-3′ and 5′-GGACTACHVGGGTWTCTAAT-3′) and ITS5/ITS2 (5′-GGAAGTAAAAGTCGTAACAAGG-3′ and 5′-GCT GCGTTCTTCATCGATGC-3′). Agencourt AMPure Beads (Beckman Coulter, Indianapolis, IN, United States) and the PicoGreen dsDNA Assay kit (Invitrogen, Carlsbad, CA, United States) were used to purify and quantify the PCR amplicons, respectively. After quantification of individual amplicons, equal amounts of barcoded amplicons were pooled, and paired-end 2 × 300 bp reads were obtained by sequencing on the Illumina MiSeq platform at Shanghai Personal Biotechnology Co., Ltd. (Shanghai, China). The raw sequencing data were deposited in the National Center for Biotechnology Information Sequence Read Archive with accession no. PRJNA542035. The *B. minax* adult gut 16s rRNA data was from Wang et al. (2014) (under accession number SRR1531158 in NCBI).

### Sequence Analysis and Diversity Measures

Sequencing data were processed by the Quantitative Insights into Microbial Ecology (QIIME, v1.8.0) pipeline (Caporaso et al., 2010). After filtering low-quality sequences according to previous criteria (Gill et al., 2006), FLASH was employed to assemble the paired-end reads (Magoč and Salzberg, 2011). After chimera detection, all remaining high-quality sequences were clustered into operational taxonomic units (OTUs) with a similarity threshold of 97% sequence identity by UCLUST (Edgar, 2010). The Greengenes Database (DeSantis et al., 2006) and the Unite Database (Kõljalg et al., 2013) were used for BLAST searching with the representative sequences and further taxonomy classification.

The qualified OTU data in QIIME were used to calculate the alpha diversity indices, including the Chao1 richness estimator, the abundance-based coverage estimator (ACE) metric and the Shannon and Simpson diversity index. Regardless of the relative abundance and based only on the occurrence of OTUs, the R package “VennDiagram” was used to generate a Venn diagram to visualize shared and unique OTUs across the lifespan of larvae (Zaura et al., 2009). Beta diversity analysis was employed to assess the structural variation of microbial communities among samples based on UniFrac distance metrics (Lozupone and Knight, 2005; Lozupone et al., 2007) visualized via non-metric multidimensional scaling (NMDS) and unweighted pair-group method with arithmetic means (UPGMA) hierarchical clustering (Ramette, 2007). Phylogenetic investigation of communities by reconstruction of unobserved states (PICRUSt) was performed to predict microbial functions using the high-quality sequences (Langille et al., 2013).

### Statistical Analysis

Student’s *t*-test and one-way analysis of variance by Tukey’s test were used to calculate the statistical significance among different groups, and a *P*-value < 0.05 was representative of statistical significance. Permutational multivariate analysis of variance (PERMANOVA) was performed to assess the differences in the microbiota structure among groups (Mcardle and Anderson, 2001). The fitting curve of the microbiota, Spearman correlations and correlation heatmaps were performed with R statistical software^1^. GraphPad Prism 8.0 (GraphPad Software, La Jolla, CA, United States) and R statistical software were used to generate all the diagrams in the present study.

## Results

### Composition of Gut Microbiota Across the Life Stages of *B. minax* Larvae

High-throughput sequencing was employed to investigate the composition of the bacterial and fungal communities that inhabit the larval gut. The sequencing data yielded a total of 930,502 reads of bacterial 16S rRNA genes and 1,058,828 reads of the ITS region of fungal rRNA genes with average lengths of 435 and 202 bp, respectively. Species accumulation curve analysis indicated that the samples were sufficient to reveal the microbial communities, as the species accumulation curves tended toward saturation (Supplementary Figures S1A, S2A). The rank-abundance curves showed that most of the reads from the bacterial and fungal communities belonged to rare species and that the distribution of species was relatively concentrated according to the breadth and smoothness of the curves (Supplementary Figures S1B, S2B).

**FIGURE 1:**
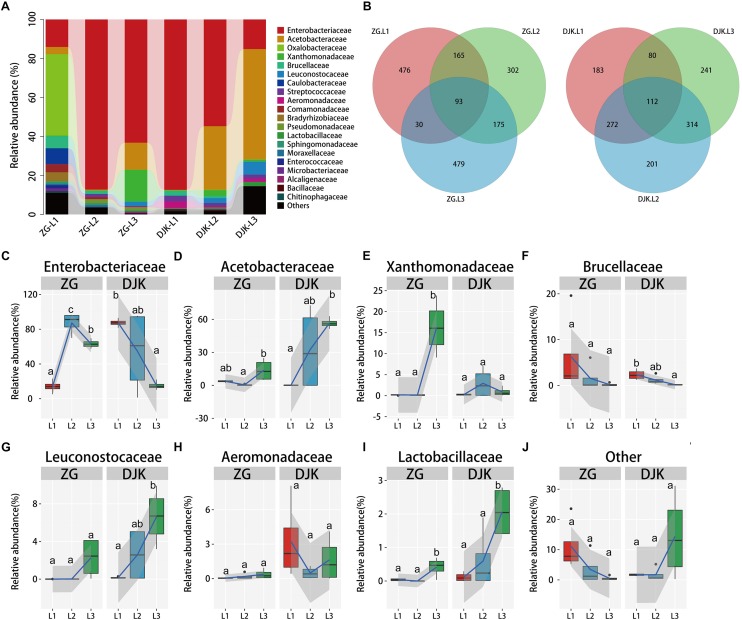
Relative abundance of bacterial families across the lifespan of larvae. **(A)** Taxonomic breakdown at the family level grouped by different populations with different development stages. **(B)** Overlap of OTUs at the larval stage. **(C–J)** Boxplots showing the mean abundance of core bacteria in the larval gut. In every panel, the left side represents the population collected from Zigui (ZG, *n* = 4), and the right side represents the population collected from Danjiangkou (DJK, *n* = 4). The high, low and median data values are shown in boxplots, and the lower and upper edges of every box represent the first and third quartiles, respectively. Multiple comparisons were performed in the two groups separately. Different letters indicate a significant difference between different instars in each group (*p* < 0.05, one-way ANOVA, Tukey *post hoc* test). The curve fitting was created using the “ggplot2” package in R statistical analyses. The lines show median values per region window, and the shaded area denotes the estimated 95% confidence interval.

Taxonomic analysis of gut bacteria revealed that all samples were intimately associated with diverse microbes across the lifespan of larvae (Figure 1A). The most prevalent phyla were Proteobacteria and Firmicutes, and they were detected in all larvae of *B. minax* from two field populations (Supplementary Figure S3). Venn diagram analysis showed that subsets of 93 and 112 shared bacterial OTUs resided in the gut across the lifespan of larvae from ZG and DJK, respectively (Figure 1B). Most of the shared OTUs belonged to Enterobacteriaceae (Supplementary Data Sheet S1). Enterobacteriaceae were the core members of gut bacteria communities in both larvae (Figure 1C) and adults of *B. minax* (Supplementary Table S1). With the development of larvae, there was a significant increase in the relative abundance of Acetobacteraceae, Leuconostocaceae, and Lactobacillaceae (Figures 1D,G,I), while the relative abundance of bacteria of these families was different in larvae at the same instar stage from different populations, especially 3rd instar larvae (Supplementary Table S1). By contrast, we found some bacterial families, including Brucellaceae, Chitinophagaceae, Moraxellaceae, and Bacillaceae, to be decreased or absent in both field populations during the development of larvae (Figure 1F and Supplementary Figures S4I–K). The relative abundance of some bacterial families, including Oxalobacteraceae, Comamonadaceae, Caulobacteraceae, Enterococcaceae, Bradyrhizobiaceae, and Sphingomonadaceae, only existed in the gut of 1st or 2nd instar from ZG, and the abundance decreased to undetectable levels in 3rd instar larvae (Supplementary Figure S4). We also found a few bacteria, such as Xanthomonadaceae, varied between the two groups, and the abundance of these bacteria sharply increased in 3rd instar larvae from ZG but not from DJK (Figure 1E). Pseudomonadaceae consistently survived in the gut of larvae collected from ZG (Supplementary Figure S4D), while Aeromonadaceae and Streptococcaceae stably resided in the gut of larvae collected from DJK (Figure 1H and Supplementary Figure S4E). Moreover, there was a sharp decrease in the relative abundance of other bacteria (unclassified and very low abundance bacteria) in ZG samples during larval development, but the relative abundance of other bacteria was stable and persisted in the gut of larvae collected from DJK (Figure 1J).

**FIGURE 2:**
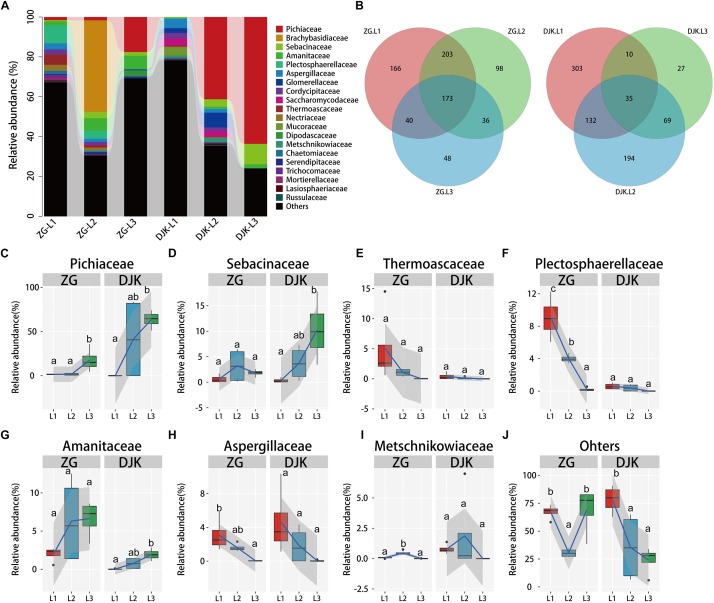
Relative abundance of fungi across the lifespan of larvae. **(A)** Taxonomic breakdown at the family level grouped by different populations with different development stages. **(B)** Overlap of OTUs at the larval stage. **(C–J)** Boxplots showing the mean abundance of core fungi in the larval gut. In every panel, the left side represents the population collected from ZG (*n* = 4), and the right side represents the population collected from DJK (*n* = 4). The high, low, and median data values are shown in boxplots, and the lower and upper edges of every box represent the first and third quartiles, respectively. Multiple comparisons were performed in the two groups separately. Different letters indicate a significant difference between different instars in each group (*p* < 0.05, one-way ANOVA, Tukey *post hoc* test). The lines show median values per region window, and the shaded area denotes the estimated 95% confidence interval.

We further investigated the fungal composition of larval guts collected from ZG and DJK. More than 85% of the fungal sequences were annotated as belonging to the two phyla Ascomycota and Basidiomycota, which were dominant in the fungal community (Supplementary Figure S3B). There were subsets of 173 and of 35 shared fungal OTUs in the gut across the lifespan of larvae from ZG and DJK, respectively, as revealed by Venn diagram analysis (Figure 2B). At the family level, an increase of Pichiaceae, Sebacinaceae, and Amanitaceae occurred during the development of larvae in both groups, and these fungi stably resided in the gut of 3rd instar larvae (Figures 2A,C,D,G). In addition, Pichiaceae were present across three larval stages (Supplementary Data Sheet S1). During larval development, several fungi, such as Thermoascaceae, Plectosphaerellaceae, Aspergillaceae, Metschnikowiaceae, Lasiosphaeriaceae, Cordycipitaceae, Trichocomaceae, Nectriaceae, Chaetomiaceae, Mortierellaceae, and Russulaceae, decreased or disappeared in both groups (Figures 2E,F,H,I and Supplementary Figures S5F–L). However, some fungi, e.g., Glomerellaceae, Mucoraceae and Saccharomycodaceae, were only observed in the gut of 1st or 2nd instar larvae collected from DJK (Supplementary Figures S5B,C,E), Brachybasidiaceae and Dipodascaceae were only detected in the gut of the 2nd or 3rd instar larval population collected from ZG (Supplementary Figures S5A,D). There was a sharp decrease of other fungi (unclassified and very low abundance fungus) in DJK samples as larval development progressed, but the unclassified and very low abundance fungi stably resided in the gut of larvae collected from ZG (Figure 2J).

### Diversity of Gut Microbiota Across the Life Stages of *B. minax* Larvae

**FIGURE 3:**
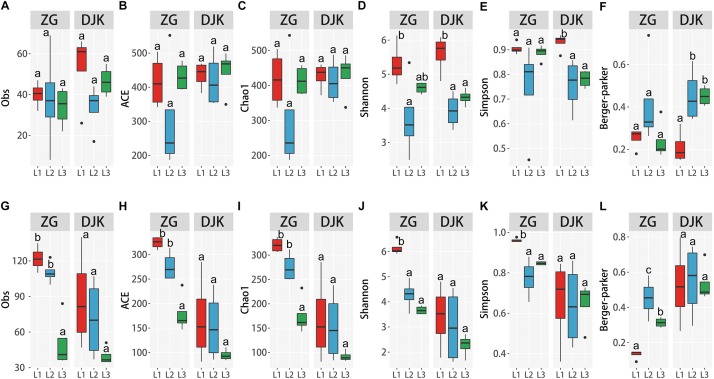
Alpha diversity of microbiota across different development stages of larvae. **(A–F)** Alpha diversity of bacteria among the three development stages of larvae according to the Obs, ACE, Chao1 and Shannon, Simpson and Berger-Parker index (*n* = 4). **(G–L)** Alpha diversity of fungi among the three development stages of larvae, as measured as Obs, ACE, Chao1, Shannon, Simpson and Berger-Parker index (*n* = 4). Multiple comparisons were performed in the two groups separately. Different letters indicate a significant difference between different instars in each group (*p* < 0.05, one-way ANOVA, Tukey *post hoc* test).

The species richness of bacteria among different life stages of larvae was analyzed using the observed species (Obs), ACE, and Chao1. Diversity was analyzed using the Shannon, Simpson and Berger-Parker index. The OTUs and two richness estimators (ACE and Chao1) showed that there were no significant differences among the different development stages of larvae in either field population (Figures 3A–C). When taking into account the diversity index (Shannon index), 1st instar larvae had the greatest species diversity in both groups, and there was a decrease in species diversity with the development of larvae (Figure 3D). While only the Simpson and Berger-Parker estimators of larvae collected from DJK displayed the same pattern as above, there were no significant differences across the lifespan of larvae collected from ZG based on the Simpson and Berger-Parker estimator (Figures 3E,F).

Analysis of the alpha diversity of fungi among the different life stages of larvae demonstrated that during development, there was a significant decrease in species richness and diversity of fungi in the gut of larvae collected from ZG based on Obs, ACE, Chao1, and the Shannon, Simpson and Berger-Parker index (Figures 3G–L). First instar larvae had the greatest fungal richness and species diversity. Linear curve analysis of alpha diversity showed that there was also a decrease in species richness and diversity of fungi as larvae collected from DJK developed from 1st instar to 3rd instar (Figures 3G–L), but this decrease was not significant.

### Structure of Gut Microbiota Across the Life Stages of *B. minax* Larvae

**FIGURE 4:**
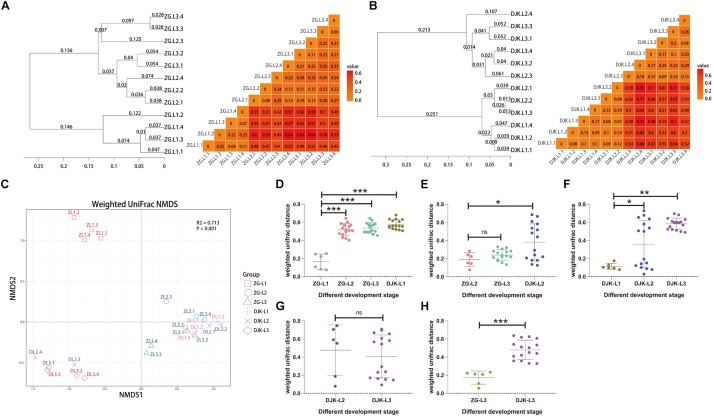
Bacterial community dissimilarity of two field populations based on UniFrac metrics. **(A)** UPGMA (left) and heatmap (right) analyses of the bacterial community in the ZG population. **(B)** UPGMA (left) and heatmap (right) analyses of the bacterial community in the DJK population. **(C)** NMDS analyses of all samples. Each symbol represents a sample, and different colors represent the different development stages of larvae. Variation in communities segregated strongly according to host developmental stage and different field populations (PERMANOVA test with 999 permutations, *P* ≤ 0.001). **(D–H)** Weighted UniFrac distances between different development stages (*n* = 6∼16). In each panel, the first entry in the X axis serves as the reference for the comparisons, and the median is plotted as the horizontal line. ^∗^*p* < 0.05; ^∗∗^*p* < 0.01; and ^∗∗∗^*p* < 0.001; ns, not significant as calculated by Student’s *t*-test.

Based on UniFrac metrics, the bacterial, and fungal community dissimilarity was investigated. UPGMA and heatmap analyses showed that there was an apparent separation between early instar (1st instar larvae) and late instar (3rd instar larvae) larvae in both field populations, and larvae from the same instar were tightly clustered (Figures 4A,B). Although 2nd instar larvae clustered with the 1st and 3rd instar larval groups, there was a significant difference in the UniFrac distance between 1st instar and 2nd instar larvae (Figures 4D,F) but no difference between 2nd instar and 3rd instar larvae (Figures 4E,G), indicating that the structure of 2nd instar larval gut bacteria is similar to that of 3rd instar larvae. NMDS analyses also revealed that the composition and structure of the gut bacterial community varied among the different life stages of larvae in both field populations. In addition, NMDS was sufficient to separate larvae of different stages and the same instar larval stage from different field populations (PERMANOVA test with 999 permutations, *P* < 0.001) (Figures 4C–E,H).

**FIGURE 5:**
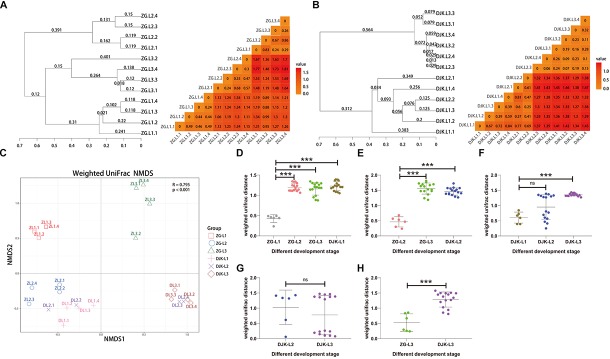
Fungal community dissimilarity of two field populations based on UniFrac metrics. **(A)** UPGMA (left) and heatmap (right) analyses of the fungal community in the ZG population. **(B)** UPGMA (left) and heatmap (right) analyses of the fungal community in the DJK population. **(C)** NMDS analyses of all samples. Each symbol represents a sample, and different colors represent different development stages of larvae. Variation in communities segregated strongly according to host development stage and different field populations (PERMANOVA test with 999 permutations, *P* ≤ 0.001). **(D–H)** Weighted UniFrac distances between different development stages (*n* = 6∼16). In each panel, the first entry in the X axis serves as the reference for the comparisons, and the median is plotted as a horizontal line. ^∗∗∗^*p* < 0.001; ns, not significant as calculated by Student’s *t*-test.

We next analyzed fungal community dissimilarity across the lifespan of larvae. UPGMA and heatmap analyses showed that there was a relatively tight clustering of the fungal communities of larvae at the same instar stage (Figure 5A) and that the UniFrac distances among different larval stages were significantly different in the ZG population (Figures 5D,E). There was also an apparent separation between early instar and late instar larvae in the DJK population (Figure 5B), with a significant difference in UniFrac distance between 1st instar and 3rd instar larvae (Figure 5F). While the 2nd instar larvae clustered with 1st and 3rd instar larval groups, there was no significant difference in the UniFrac distance between 2nd instar larvae and other development stages (Figures 5F,G). NMDS analyses also revealed that the composition and structure of the gut mycobiota varied among the different life stages of larvae in both field populations and was sufficient to differentiate the various larval stages and same instar larval stage from different populations (PERMANOVA test with 999 permutations, *P* < 0.001) but not from 2nd instar larvae in DJK (Figures 5C–E,H).

### PICRUSt Metagenomic Predictions of Microbial Communities in Larval Gut

**FIGURE 6:**
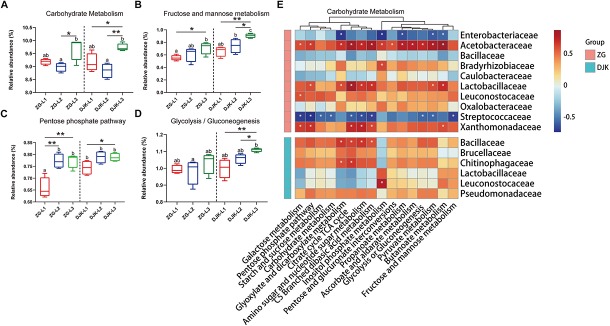
Comparison of predicted KEGG pathways of larvae and Spearman correlation analysis. The inferred metabolic pathways are shown with carbohydrate metabolism **(A)**, fructose and mannose metabolism **(B)**, pentose phosphate pathway **(C)**, and glycolysis/gluconeogenesis **(D)**. Spearman correlation analysis between the carbohydrate metabolic pathways and bacteria (taxonomic breakdown at the family level) that vary significantly during the development of larvae is presented in heatmaps **(E)**. Multiple comparisons were performed with one-way analysis of variance and Tukey’s *post hoc* test. Different letters indicate a significant difference between different samples (*p* < 0.05). ^∗^*p* < 0.05 and ^∗∗^*p* < 0.01, calculated by Student’s *t*-test. In heatmaps, blue represents negative correlation between gene expression and bacterial genus, and red represents a positive correlation, ^∗^*P* < 0.05.

Approximately 41 KEGG pathways were obtained using PICRUSt to infer the functions of the bacterial communities (Supplementary Data Sheet S2). The nearest sequenced taxon index (NSTI) of larval samples is shown in Supplementary Table S2. Here, we mainly focused on the metabolic pathways that had a >1% relative abundance. The dynamic change patterns of most metabolic pathways across the development stages of larvae were different in the two field populations (Supplementary Figure S6) except for carbohydrate metabolism, which was increased across the lifespan of larvae in both groups (Figure 6A). Within carbohydrate metabolism, we found that several pathways were also increased with the development of larvae, such as fructose and mannose metabolism, pentose phosphate pathway, and glycolysis or gluconeogenesis (Figures 6B–D). The relative abundances of these metabolic pathways in larvae from DJK were higher than in the corresponding instar larvae from ZG (Figures 6B–D). To clarify the association between functionality and the changes in the composition of the bacterial microbiota, Spearman correlation was employed. The results showed that the Acetobacteraceae, Lactobacillaceae, Leuconostocaceae, and Xanthomonadaceae had positive correlations with almost all the predicted pathways of carbohydrate metabolism in ZG samples and that Lactobacillaceae and Leuconostocaceae displayed positive correlations with butanoate metabolism, fructose and mannose metabolism and inositol phosphate metabolism in DJK samples (Figure 6E).

## Discussion

Here, we surveyed the microbiota of *B. minax* larvae from two different field populations, and all the samples were intimately associated with a wide variety of microbes. Similar to other insects, we found that Proteobacteria and Firmicutes dominated the bacterial community across the lifespan of larvae (Yun et al., 2014; Chen et al., 2018). For the first time, we also investigated the mycobiota of the *B. minax* larval gut, and Ascomycota and Basidiomycota were found to constitute the main members of the fungal community.

Cluster analysis showed a clear separation of different instar larval gut microbiotas, with the exception of 2nd instar larvae, which clustered with early or late instar larval groups because 2nd instar larvae were in a developmental transition from 1st instar to 3rd instar larvae. Additionally, there was also a significant separation of the microbiota community composition in the same instar larval stage collected from different locations. The microbiota of the insect gut is influenced by several factors, such as the environmental habitat, diet, immune system, physiology, and phylogeny of host (Yun et al., 2014; Yao et al., 2016; Martinson et al., 2017; Mistry et al., 2017). Considering that larvae of both populations were developing in different hosts and locations, it is highly likely that the diet and environment played a role in shaping the microbial community structure of larvae. Although the structure of microbiota was different within the two *B. minax* field populations, a similar shift pattern across the life stages of larvae was identified. This corresponding dynamic change in the gut microbiota during the host development indicates that host physiology may play a vital role in determining the microbiota composition.

Across the different larval developmental stages, we found that only a few bacteria stably resided in the gut of 3rd instar larvae. Most bacterial community members belonged to Enterobacteriaceae, Acetobacteraceae, Lactobacillaceae, and Leuconostocaceae families. Enterobacteriaceae is also the dominant commensal bacterial family in the gut and reproductive organ of *B. minax* adults (Wang et al., 2014) and in the gut of several other tephritids, including *Anastrepha*, *Ceratitis*, and *Dacus* (Drew and Lloyd, 1991; Kuzina et al., 2001; Behar et al., 2008). Enterobacteriaceae bacterial family have been reported to participate in nitrogen fixation and pectinolysis (Behar et al., 2005, 2008). Recently, Zhao et al. (2018) found that Enterobacteriaceae, Acetobacteraceae, and Lactobacillaceae were the main families in *B. dorsalis* larvae and likely participated in sugar metabolism. Previously study showed that bacteria in Acetobacteraceae and Lactobacillaceae families played a vital role in *Drosophila* larval development by modulating sugar metabolism related insulin pathways (Shin et al., 2011; Storelli et al., 2011). *Leuconostoc*, *Fructobacillus*, *Oenococcus*, and *Weissella* are representative genera in Leuconostocaceae family. It has been reported that *Leuconostoc* species can ferment fructose (Ljungdahl, 1962). A specific group of bacteria that solely inhabit fructose-rich niches has been characterized and defined as fructophilic lactic acid bacteria (FLAB) (Endo and Salminen, 2013). FLAB have been found in the guts of bees, tropical fruit flies and giant ants, which live by feeding on flowers or fructose diet (Thaochan et al., 2010; He et al., 2011; Koch and Schmid-Hempel, 2011). It is well known that the sugar content increases as a fruit matures (Chen et al., 2012). Following the oviposition of *B. minax*, a ripening process begins in the fruit, resulting in sugar-rich fruit (Fischer et al., 2016). This high sugar content provides a favorite substrate for these bacteria to colonize and can be fermented by larvae-associated gut microbiota. In our study, the dynamic increases of three important bacterial families Acetobacteraceae, Lactobacillaceae, and Leuconostocaceae were consistent with the PICRUSt prediction of increased carbohydrate metabolism pathways. Spearman correlation analysis further showed that these three bacterial families displayed positive correlations with carbohydrate metabolism pathways, suggesting that these microbial families can help the host to remarkably adapt to this environment, digest fruit, and provide amino acids or other nutrients. Of note, the relative abundances of these three bacterial families were different in the same instar larval stage developed in different plant hosts, higher in DJK than in ZG samples (Supplementary Table S1). This difference in bacterial abundance may explain why some metabolic pathways, such as fructose and mannose metabolism, were more active in DJK larvae than in ZG. It is plausible that the difference in bacterial abundance was influenced by the presence of different metabolic components in orange and mandarin orange, such as the sugar-acid ratio in fruit. In future, it would be interesting to uncover the relationship among the insect, its gut microbiota and its plant host.

There was also a sharp decrease in the relative abundances of several bacterial families during the development of larvae, including Brucellaceae, Chitinophagaceae, Moraxellaceae, and Bacillaceae. These bacterial families may represent transient microbes or may perform their function at a special development stage of larvae. Oxalobacteraceae, Comamonadaceae, Caulobacteraceae, Enterococcaceae, Bradyrhizobiaceae, and Sphingomonadaceae were only detected in the gut of 1st or 2nd instar larvae from ZG, and they decreased to an undetectable level in 3rd instar larvae; thus, it is plausible that environmental microbes that originate from the egg and native plant diet will be specially assembled in newly emerged larvae (Chen et al., 2018). This large difference of early instar larvae microbiota may explain why the dynamic change patterns of most metabolic pathways across the development stages of larvae were different between the two field populations.

Most research to elucidate insect-microbiota interactions has solely focused on bacteria. However, fungus, as an important member of this interaction relationship, has largely been overlooked (Broderick and Lemaitre, 2012). In the present study, Ascomycota and Basidiomycota were dominant in the mycobiota of *B. minax* larvae. Several orders of insects are intimately associated with yeasts, including flies, bees, beetles and ants (Becher et al., 2012; Vogel et al., 2017), and most of these associated yeasts belong to the Ascomycota phylum (Chandler et al., 2012). The functions of yeast have largely been studied in *Drosophila*. Yeasts are vital for larval development because they provide the host with vitamins, sterols and amino acids (Becher et al., 2012; Broderick and Lemaitre, 2012; Yamada et al., 2015) and can attract *Drosophila* via ester production (Christiaens et al., 2014), inducing *Drosophila* egg-laying behavior (Becher et al., 2012, Fischer et al., 2016). More recently, Fischer et al. (2016) demonstrated that *Drosophila* prefers a *Saccharomyces*-*Acetobacter* co-culture to the same microorganisms grown separately, and *Acetobacter* metabolism of ethanol derived from *Saccharomyces* fungus was essential for this co-culture preference, indicating that the coexistence of this bacterial, and fungal microbiota relationship is beneficial for the host. In our study, we found that there was a sharp increase in Pichiaceae yeast in accordance with Acetobacteraceae bacteria. The functions of Pichiaceae yeast and whether there is a bacteria-fungus interaction within the microbiome of *B. minax* larvae, as has been shown in *Drosophila*, remain unknown. Strikingly, there was also a sharp decrease in the relative abundances of several fungi, including Thermoascaceae, Plectosphaerellaceae, Aspergillaceae, Metschnikowiaceae, Lasiosphaeriaceae, Cordycipitaceae, Trichocomaceae, Nectriaceae, Chaetomiaceae, Mortierellaceae, and Russulaceae. These fungi may represent transient microbes or opportunistic pathogenic fungi of citrus. *B. minax* larvae develop within rotting fruit that may contain these environment fungi, while host metabolites, such as acetic acid, or gut-associated microbes may inhibit these heterotrophic microorganisms during larval development (Kang et al., 2003).

## Conclusion

In conclusion, the present study demonstrated that the gut microbiota of *B. minax* larvae greatly differ across different development stages and that several members of microbiota exhibit similar shift patterns in both field populations, which may be indispensable to their adaption to the environment and development of the host. More research is required to explore host-microbe interactions in *B. minax*. These findings will advance the understanding of the gut microbiota of this harmful citrus pest and may provide clues to develop novel biocontrol strategies against *B. minax*.

## Data Availability Statement

The datasets generated for this study can be found in the PRJNA542035.

## Author Contributions

HZ and ZY conceived and designed the project. ZY performed the experiments and analyzed the data. ZY and ZC generated the graphs. QM, SB, YW, PZ, and HM collected the samples and extracted the DNA. HZ, ZY, QM, and MR wrote the manuscript.

## Conflict of Interest

The authors declare that the research was conducted in the absence of any commercial or financial relationships that could be construed as a potential conflict of interest.

## Footnotes

1 http://cran.r-project.org/

## Supplementary Material

FIGURE S1 Species accumulation and rank-abundance curves of bacteria based on bacterial OTUs at a similarity level of 97%. **(A)** Species accumulation curves. **(B)** Rank-abundance curves.

FIGURE S2 Species accumulation and rank-abundance curves of fungus based on bacterial OTUs at a similarity level of 97%. **(A)** Species accumulation curves. **(B)** Rank-abundance curves.

FIGURE S3 Taxonomic compositions of microbiotas at the phylum level. **(A)** Bacterial composition of two field populations. **(B)** Fungal composition of two field populations.

FIGURE S4 Relative abundance of bacterial families across the lifespan of larvae. **(A–K)** Boxplots showing the mean abundance of bacteria in the larval gut. In every panel, the left side represents the population collected from ZG (*n* = 4), and the right side represents the population collected from DJK (*n* = 4). Multiple comparisons were performed in the two group separately. Different letters indicate a significant difference between different instars in each group (*p* < 0.05, one-way ANOVA, Tukey *post hoc* test). The lines show median values per region window, and the shaded area denotes the estimated 95% confidence interval.

FIGURE S5 Relative abundance of fungi across the lifespan of larvae. **(A–L)** Boxplots showing the mean abundance of fungi in the larval gut. In every panel, the left side represents the population collected from ZG (*n* = 4), and the right side represents the population collected from DJK (*n* = 4). Multiple comparisons were performed in the two group separately. Different letters indicate a significant difference between different instars in each group (*p* < 0.05, one-way ANOVA, Tukey *post hoc* test). The lines show median values per region window, and the shaded area denotes the estimated 95% confidence interval.

FIGURE S6 Predicted gut bacterial functions of larvae from two field populations. **(A–J)** the inferred metabolic pathways amino acid metabolism, energy metabolism, metabolism of cofactors and vitamins, xenobiotics biodegradation and metabolism, lipid metabolism, nucleotide metabolism, glycan biosynthesis and metabolism, enzyme families, metabolism of other amino acids and metabolism of terpenoids and polyketides are shown at the second hierarchical level (*n* = 4). Multiple comparisons were performed in the two group separately. Different letters indicate a significant difference between different instars in each group (*p* < 0.05, one-way ANOVA, Tukey *post hoc* test).

TABLE S1 Comparisons of the relative abundance of gut bacteria from larval and adult samples at family level.

TABLE S2 The nearest sequenced taxon index (NSTI) of larval samples.

DATA SHEET S1 The shared OTUs across the lifespan of larvae.

DATA SHEET S2 The relative abundance of KEGG pathways in larvae predicted by PICRUSt.

## References

[B1] Becher P. G. Flick G. Rozpędowska E. Schmidt A. Hagman A. Lebreton S. (2012). Yeast, not fruit volatiles mediate *Drosophila melanogaster* attraction, oviposition and development. *Funct. Ecol.* 26 822–828. 10.1111/j.1365-2435.2012.02006.x

[B2] Behar A. Jurkevitch E. Yuval B. (2008). Bringing back the fruit into fruit fly-bacteria interactions. *Mol. Ecol.* 17 1375–1386. 10.1111/j.1365-294X.2008.03674.x 18302695

[B3] Behar A. Yuval B. Jurkevitch E. (2005). Enterobacteria mediated nitrogen fixation in natural populations of the fruit fly *Ceratitis capitata*. *Mol. Ecol.* 14 2637–2643. 10.1111/j.1365-294X.2005.02615.x 16029466

[B4] Broderick N. A. Lemaitre B. (2012). Gut-associated microbes of *Drosophila melanogaster*. *Gut Microbes* 3 307–321. 10.4161/gmic.19896 22572876 PMC3463489

[B5] Broderick N. A. Raffa K. F. Goodman R. M. Handelsman J. (2004). Census of the bacterial community of the gypsy moth larval midgut by using culturing and culture-independent methods. *Appl. Environ. Microbiol.* 70 293–300. 10.1128/AEM.70.1.293-300.2004 14711655 PMC321235

[B6] Brune A. Dietrich C. (2015). The gut microbiota of termites: digesting the diversity in the light of ecology and evolution. *Annu. Rev. Microbiol.* 69 145–166. 10.1146/annurev-micro-092412-155715 26195303

[B7] Cai Z. Yao Z. Li Y. Xi Z. Bourtzis K. Zhao Z. (2018). Intestinal probiotics restore the ecological fitness decline of *Bactrocera dorsalis* by irradiation. *Evol. Appl.* 11 1946–1963. 10.1111/eva.12698 30459840 PMC6231467

[B8] Caporaso J. G. Kuczynski J. Stombaugh J. Bittinger K. Bushman F. D. Costello E. K. (2010). QIIME allows analysis of high-throughput community sequencing data. *Nat. Methods* 7 335–336. 10.1038/nmeth.f.303 20383131 PMC3156573

[B9] Ceja-Navarro J. A. Vega F. E. Karaoz U. Hao Z. Jenkins S. Lim H. C. (2015). Gut microbiota mediate caffeine detoxification in the primary insect pest of coffee. *Nat. Commun.* 6:7618. 10.1038/ncomms8618 26173063 PMC4510693

[B10] Chandler J. A. Eisen J. A. Kopp A. (2012). Yeast communities of diverse drosophila species: comparison of two symbiont groups in the same hosts. *Appl. Environ. Microbiol.* 78 7327–7336. 10.1128/AEM.01741-12 22885750 PMC3457106

[B11] Chen B. Du K. Sun C. Vimalanathan A. Liang X. Li Y. (2018). Gut bacterial and fungal communities of the domesticated silkworm (*Bombyx mori*) and wild mulberry-feeding relatives. *ISME. J.* 12 2252–2262. 10.1038/s41396-018-0174-1 29895989 PMC6092317

[B12] Chen M. Jiang Q. Yin X. R. Lin Q. Chen K. S. (2012). Effect of hot air treatment on organic acid- and sugar-metabolism in ponkan (*Citrus reticulata*) fruit. *Sci. Hortic.* 147 118–125. 10.1016/j.scienta.2012.09.011

[B13] Chen Z. Dong Y. Wang Y. Andongma A. A. Rashid M. A. Krutmuang P. (2016). Pupal diapause termination in *Bactrocera minax*: an insight on 20-hydroxyecdysone induced phenotypic and genotypic expressions. *Sci. Rep.* 6:27440. 10.1038/srep27440 27273028 PMC4897610

[B14] Christiaens J. F. Franco L. M. Cools T. L. De Meester L. Michiels J. Wenseleers T. (2014). The fungal aroma gene ATF1 promotes dispersal of yeast cells through insect vectors. *Cell Rep.* 9 425–432. 10.1016/j.celrep.2014.09.009 25310977

[B15] DeSantis T. Z. Hugenholtz P. Larsen N. Rojas M. Brodie E. L. Keller K. (2006). Greengenes, a chimera-checked 16S rRNA gene database and workbench compatible with ARB. *Appl. Environ. Microbiol.* 72 5069–5072. 10.1128/AEM.03006-05 16820507 PMC1489311

[B16] Douglas A. E. (2015). Multiorganismal insects: diversity and function of resident microorganisms. *Annu. Rev. Entomol.* 60 17–34. 10.1146/annurev-ento-010814-020822 25341109 PMC4465791

[B17] Drew R. A. Lloyd A. C. (1991). “Bacteria in the life cycle of tephritid fruit flies,” in *Microbial Mediation of Plant-herbivore Interactions*, eds Barbosa P. Krishchik V. A. Jones C. G. (New York, NY: John Wiley & Sons), 441–465.

[B18] Drew R. A. I. Dorji C. Romig M. C. Loday P. (2006). Attractiveness of various combinations of colors and shapes to females and males of *Bactrocera minax* (Diptera: Tephritidae) in a commercial mandarin grove in Bhutan. *J. Econ. Entomol.* 99 1651–1656. 10.1603/0022-0493-99.5.1651 17066795

[B19] Edgar R. C. (2010). Search and clustering orders of magnitude faster than BLAST. *Bioinformatics* 26 2460–2461. 10.1093/bioinformatics/btq461 20709691

[B20] Endo A. Salminen S. (2013). Honeybees and beehives are rich sources for fructophilic lactic acid bacteria. *Syst. Appl. Microbiol.* 36 444–448. 10.1016/j.syapm.2013.06.002 23845309

[B21] Endt K. Stecher B. Chaffron S. Slack E. Tchitchek N. Benecke A. (2010). The microbiota mediates pathogen clearance from the gut lumen after non-typhoidal *Salmonella* diarrhea. *PLoS Pathog.* 6:e1001097. 10.1371/journal.ppat.1001097 20844578 PMC2936549

[B22] Engel P. Moran N. A. (2013). The gut microbiota of insects-diversity in structure and function. *FEMS Microbiol. Rev.* 37 699–735. 10.1111/1574-6976.12025 23692388

[B23] Fischer C. N. Trautman E. P. Crawford J. M. Stabb E. V. Handelsman J. Broderick N. A. (2016). Metabolite exchange between microbiome members produces compounds that influence *Drosophila* behavior. *eLife* 6:e18855. 10.7554/eLife.18855 28068220 PMC5222558

[B24] Gill S. R. Pop M. Deboy R. T. Eckburg P. B. Turnbaugh P. J. Samuel B. S. (2006). Metagenomic analysis of the human distal gut microbiome. *Science* 312 1355–1359. 10.1126/science.1124234 16741115 PMC3027896

[B25] Grenier A. M. Duport G. Pagès S. Condemine G. Rahbé Y. (2006). The phytopathogen *Dickeya dadantii* (Erwinia chrysanthemi 3937) is a pathogen of the pea aphid. *Appl. Environ. Microbiol.* 72 1956–1965. 10.1128/AEM.72.3.1956-1965.2006 16517643 PMC1393189

[B26] He H. Chen Y. Zhang Y. Wei C. (2011). Bacteria associated with gut lumen of *Camponotus japonicus* Mayr. *Environ. Entomol.* 40 1405–1409. 10.1603/EN11157 22217755

[B27] Hongoh Y. Deevong P. Inoue T. Moriya S. Trakulnaleamsai S. Ohkuma M. (2005). Intra- and interspecific comparisons of bacterial diversity and community structure support coevolution of gut microbiota and termite host. *Appl. Environ. Microbiol.* 71 6590–6599. 10.1128/AEM.71.11.6590-6599.2005 16269686 PMC1287746

[B28] Kang H. C. Park Y. H. Go S. J. (2003). Growth inhibition of a phytopathogenic fungus, *Colletotrichum* species by acetic acid. *Microbiol. Res.* 158 321–326. 10.1078/0944-5013-00211 14717453

[B29] Koch H. Schmid-Hempel P. (2011). Bacterial communities in central European bumblebees: low diversity and high specificity. *Microb. Ecol.* 62 121–133. 10.1007/s00248-011-9854-3 21556885

[B30] Kõljalg U. Nilsson R. H. Abarenkov K. Tedersoo L. Taylor A. F. Bahram M. (2013). Towards a unified paradigm for sequence-based identification of fungi. *Mol. Ecol.* 22 5271–5277. 10.1111/mec.12481 24112409

[B31] Kuzina L. V. Peloquin J. J. Vacek D. C. Miller T. A. (2001). Isolation and identification of bacteria associated with adult laboratory Mexican fruit flies, *Anastrepha ludens* (Diptera: Tephritidae). *Curr. Microbiol.* 42 290–294. 10.1007/s002840110219 11178731

[B32] Langille M. G. Zaneveld J. Caporaso J. G. McDonald D. Knights D. Reyes J. A. (2013). Predictive functional profiling of microbial communities using 16S rRNA marker gene sequences. *Nat. Biotechnol.* 31 814–821. 10.1038/nbt.2676 23975157 PMC3819121

[B33] Ljungdahl L. (1962). Fermentation of fructose by *Leuconostoc mesenteroides*. *Biochim. Biophys. Acta.* 65 143–146. 10.1016/0006-3002(62)90159-2 13931087

[B34] Lozupone C. Knight R. (2005). UniFrac: a new phylogenetic method for comparing microbial communities. *Appl. Environ. Microbiol.* 71 8228–8235. 10.1128/AEM.71.12.8228-8235.2005 16332807 PMC1317376

[B35] Lozupone C. A. Hamady M. Kelley S. T. Knight R. (2007). Quantitative and qualitative beta diversity measures lead to different insights into factors that structure microbial communities. *Appl. Environ. Microbiol.* 73 1576–1585. 10.1128/AEM.01996-06 17220268 PMC1828774

[B36] Magoč T. Salzberg S. L. (2011). FLASH: fast length adjustment of short reads to improve genome assemblies. *Bioinformatics* 27 2957–2963. 10.1093/bioinformatics/btr507 21903629 PMC3198573

[B37] Martinson V. G. Douglas A. E. Jaenike J. (2017). Community structure of the gut microbiota in sympatric species of wild *Drosophila*. *Ecol. Lett.* 20 629–639. 10.1111/ele.12761 28371064

[B38] Mcardle B. H. Anderson M. J. (2001). Fitting multivariate models to community data: a comment on distance-based redundancy analysis. *Ecology* 82 290–297. 10.2307/2680104

[B39] Mistry R. Kounatidis I. Ligoxygakis P. (2017). Interaction between familial transmission and a constitutively active immune system shapes gut microbiota in *Drosophila melanogaster*. *Genetics* 206 889–904. 10.1534/genetics.116.190215 28413160 PMC5499193

[B40] Paniagua V. L. R. Enric F. Martin K. Monika H. Fatouros N. E. (2018). Bacterial symbionts in lepidoptera: their diversity, transmission, and impact on the host. *Front. Microbiol.* 9:556. 10.3389/fmicb.2018.00556 29636736 PMC5881003

[B41] Pinto-Tomás A. A. Sittenfeld A. Uribe-Lorío L. Chavarría F. Mora M. Janzen D. H. (2011). Comparison of midgut bacterial diversity in tropical caterpillars (Lepidoptera: Saturniidae) fed on different diets. *Environ. Entomol.* 40 1111–1122. 10.1603/EN11083 22251723

[B42] Ramette A. (2007). Multivariate analyses in microbial ecology. *FEMS Microbiol. Ecol.* 62 142–160. 10.1111/j.1574-6941.2007.00375.x 17892477 PMC2121141

[B43] Raymann K. Shaffer Z. Moran N. A. (2017). Antibiotic exposure perturbs the gut microbiota and elevates mortality in honeybees. *PLoS Biol.* 15:e2001861. 10.1371/journal.pbio.2001861 28291793 PMC5349420

[B44] Robinson C. J. Schloss P. Ramos Y. Raffa K. Handelsman J. (2010). Robustness of the bacterial community in the cabbage white butterfly larval midgut. *Microb. Ecol.* 59 199–211. 10.1007/s00248-009-9595-8 19924467 PMC2836246

[B45] Ryu J. H. Kim S. H. Lee H. Y. Bai J. Y. Nam Y. D. Bae J. W. (2008). Innate immune homeostasis by the homeobox gene caudal and commensal-gut mutualism in *Drosophila*. *Science* 319 777–782. 10.1126/science.1149357 18218863

[B46] Sharon G. Segal D. Ringo J. M. Hefetz A. Zilber-Rosenberg I. Rosenberg E. (2010). Commensal bacteria play a role in mating preference of *Drosophila melanogaster*. *Proc. Natl. Acad. Sci. U.S.A.* 107 20051–20056. 10.1073/pnas.1009906107 21041648 PMC2993361

[B47] Shin S. C. Kim S. H. You H. Kim B. Kim A. C. Lee K. A. (2011). *Drosophila* microbiome modulates host developmental and metabolic homeostasis via insulin signaling. *Science* 334 670–674. 10.1126/science.1212782 22053049

[B48] Stecher B. Hardt W. D. (2011). Mechanisms controlling pathogen colonization of the gut. *Curr. Opin. Microbiol.* 14 82–91. 10.1016/j.mib.2010.10.003 21036098

[B49] Storelli G. Defaye A. Erkosar B. Hols P. Royet J. Leulier F. (2011). *Lactobacillus plantarum* promotes *Drosophila* systemic growth by modulating hormonal signals through TOR-dependent nutrient sensing. *Cell Metab.* 14 403–414. 10.1016/j.cmet.2011.07.012 21907145

[B50] Thaochan N. Drew R. A. Hughes J. M. Vijaysegaran S. Chinajariyawong A. (2010). Alimentary tract bacteria isolated and identified with API-20E and molecular cloning techniques from Australian tropical fruit flies, *Bactrocera cacuminata* and *B. tryoni*. *J. Insect Sci.* 10:131. 10.1673/031.010.13101 20883132 PMC3016917

[B51] Vogel H. Shukla S. P. Engl T. Weiss B. Fischer R. Steiger S. (2017). The digestive and defensive basis of carcass utilization by the burying beetle and its microbiota. *Nat. Commun.* 8:15186. 10.1038/ncomms15186 28485370 PMC5436106

[B52] Wang A. Yao Z. Zheng W. Zhang H. (2014). Bacterial communities in the gut and reproductive organs of *Bactrocera minax* (Diptera: Tephritidae) based on 454 pyrosequencing. *PLoS One* 9:e106988. 10.1371/journal.pone.0106988 25215866 PMC4162550

[B53] Wang X. J. Luo L. Y. (1995). Research progress in the Chinese citrus fruit fly. *Entomol. Knowl.* 32 310–315.

[B54] Wang Y. Andongma A. A. Dong Y. Chen Z. Xu P. Ren X. (2018). Rh6 gene modulates the visual mechanism of host utilization in fruit fly *Bactrocera minax*. *Pest. Manag. Sci.* 75 1621–1629. 10.1002/ps.5278 30471178

[B55] Weiss B. L. Wang J. Aksoy S. (2011). Tsetse immune system maturation requires the presence of obligate symbionts in larvae. *PLoS Biol.* 9:e1000619. 10.1371/journal.pbio.1000619 21655301 PMC3104962

[B56] Xia Y. L. Ma X. L. Hou B. H. Ouyang G. (2018). A Review of *Bactrocera minax* (Diptera: Tephritidae) in China for the purpose of safeguarding. *Adv. Entomol.* 6 35–61. 10.4236/ae.2018.62005

[B57] Xiong K. C. Wang J. Li J. H. Deng Y. Q. Pu P. Fan H. (2016). RNA interference of a trehalose-6-phosphate synthase gene reveals its roles during larval-pupal metamorphosis in *Bactrocera minax* (Diptera: Tephritidae). *J. Insect Physiol.* 9 84–92. 10.1016/j.jinsphys.2016.07.003 27405007

[B58] Yamada R. Deshpande S. A. Bruce K. D. Mak E. M. Ja W. W. (2015). Microbes promote amino acid harvest to rescue undernutrition in *Drosophila*. *Cell Rep.* 10 865–872. 10.1016/j.celrep.2015.01.018 25683709 PMC4534362

[B59] Yao Z. Wang A. Li Y. Cai Z. Lemaitre B. Zhang H. (2016). The dual oxidase gene *BdDuox* regulates the intestinal bacterial community homeostasis of *Bactrocera dorsalis*. *ISME J.* 10 1037–1050. 10.1038/ismej.2015.202 26565723 PMC5029222

[B60] Yun J. H. Roh S. W. Whon T. W. Jung M. J. Kim M. S. Park D. S. (2014). Insect gut bacterial diversity determined by environmental habitat, diet, developmental stage, and phylogeny of host. *Appl. Environ. Microbiol.* 80 5254–5264. 10.1128/AEM.01226-14 24928884 PMC4136111

[B61] Zaura E. Keijser B. J. Huse S. M. Crielaard W. (2009). Defining the healthy "core microbiome" of oral microbial communities. *BMC Microbiol.* 9:259. 10.1186/1471-2180-9-259 20003481 PMC2805672

[B62] Zhao X. Zhang X. Chen Z. Wang Z. Lu Y. Cheng D. (2018). The Divergence in bacterial components associated with *Bactrocera dorsalis* across developmental stages. *Front. Microbiol.* 9:114. 10.3389/fmicb.2018.00114 29449838 PMC5799270

