# Supplementary figures and images for: Similar Shift Patterns in Gut Bacterial and Fungal Communities Across the Life Stages of Bactrocera minax Larvae From Two Field Populations
Source: Front Microbiol. 2019 Oct 9;10:2262. doi: 10.3389/fmicb.2019.02262 (PMC6794421; doi:10.3389/fmicb.2019.02262)

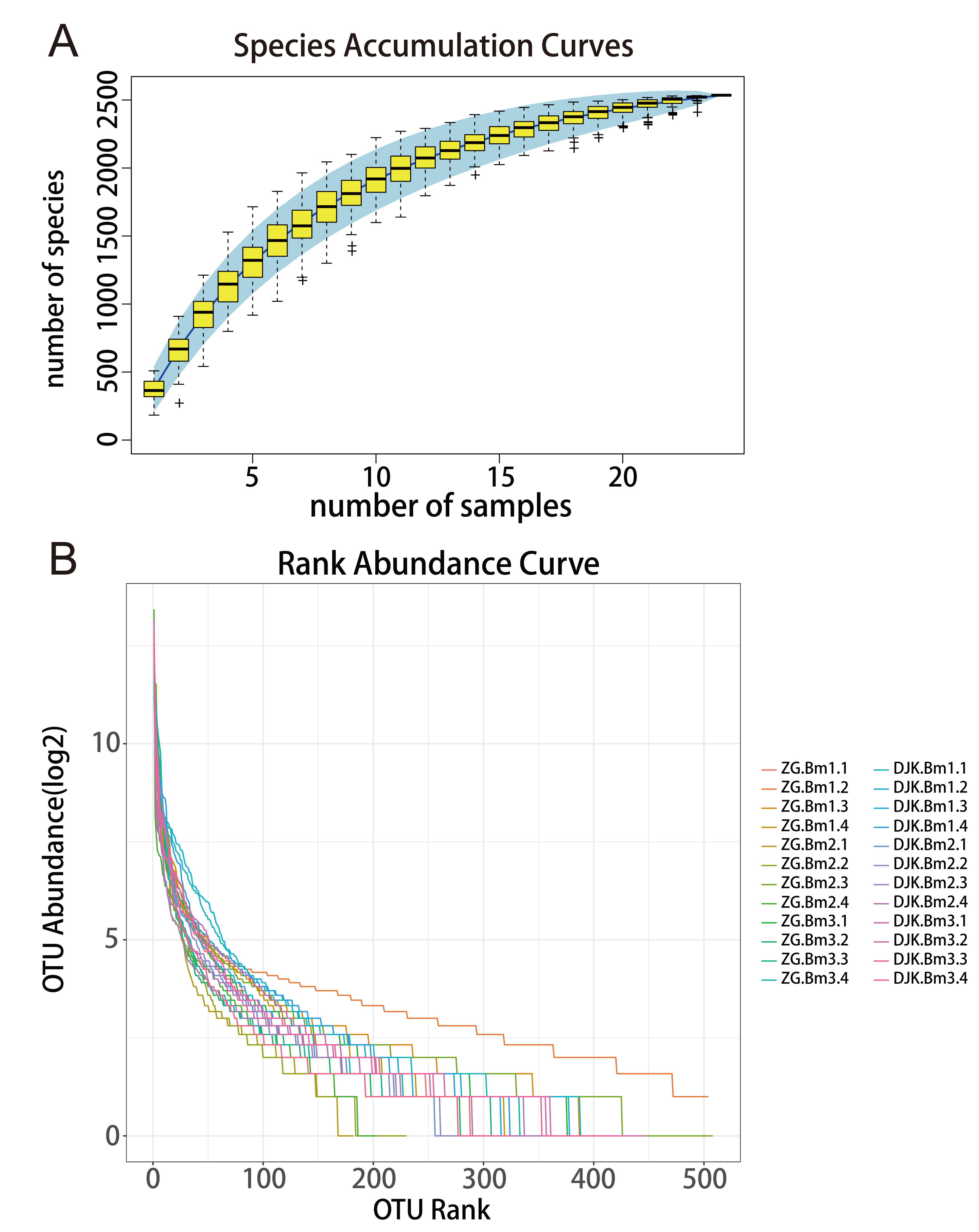

Supplement: FIGURE S1 — Species accumulation and rank-abundance curves of bacteria based on bacterial OTUs at a similarity level of 97%. (A) Species accumulation curves. (B) Rank-abundance curves. [file Image_1.JPEG]

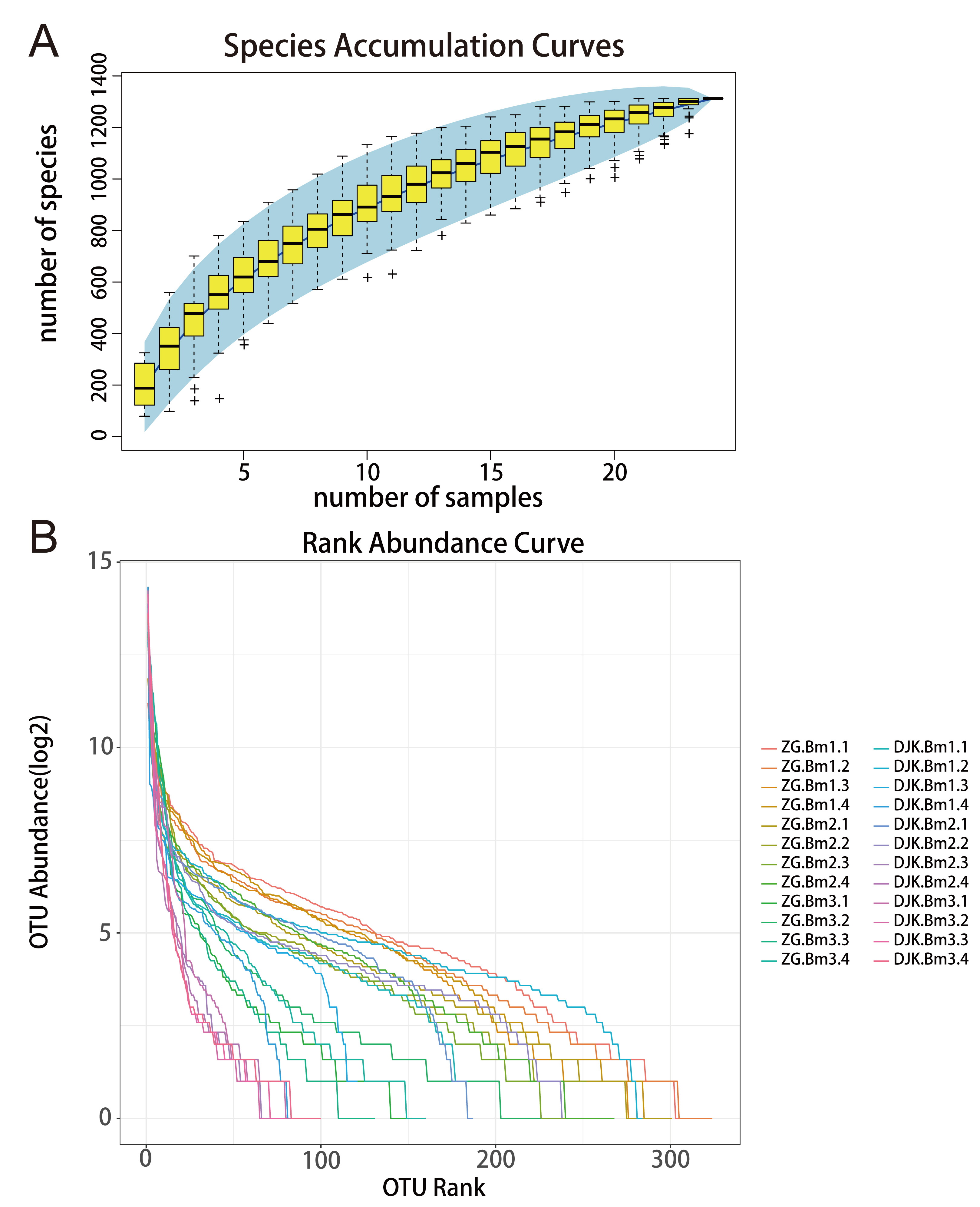

Supplement: FIGURE S2 — Species accumulation and rank-abundance curves of fungus based on bacterial OTUs at a similarity level of 97%. (A) Species accumulation curves. (B) Rank-abundance curves. [file Image_2.JPEG]

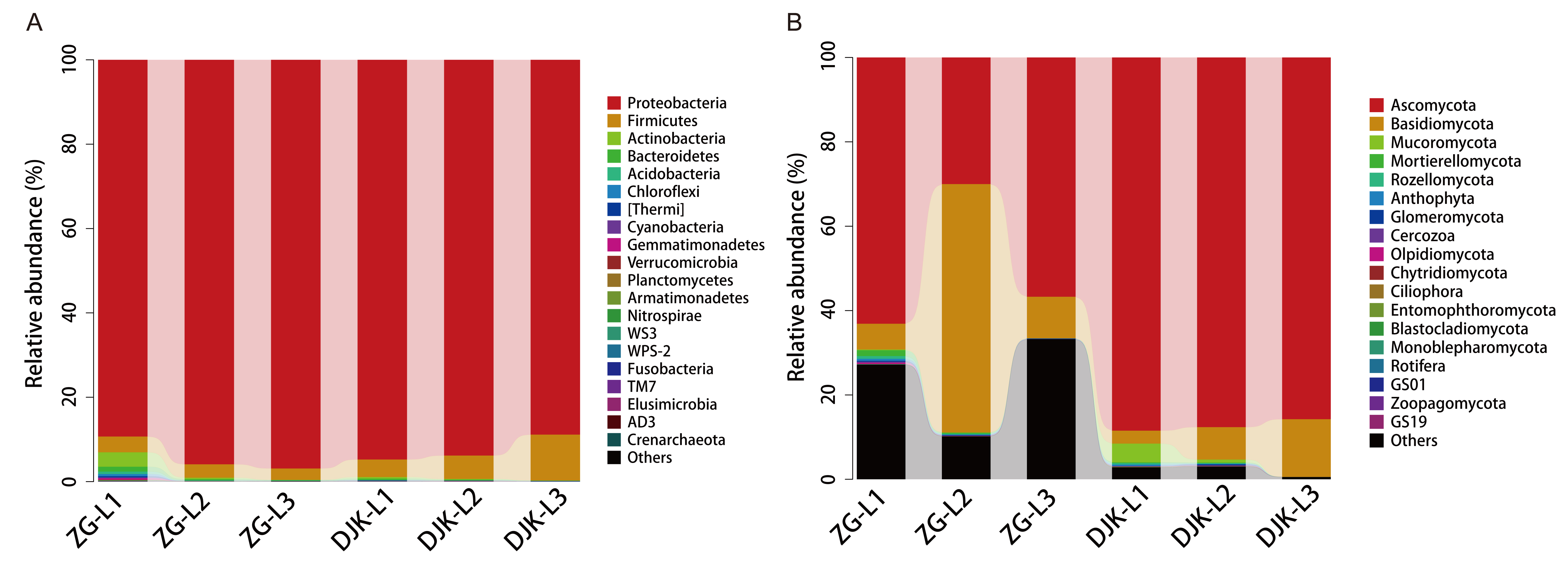

Supplement: FIGURE S3 — Taxonomic compositions of microbiotas at the phylum level. (A) Bacterial composition of two field populations. (B) Fungal composition of two field populations. [file Image_3.JPEG]

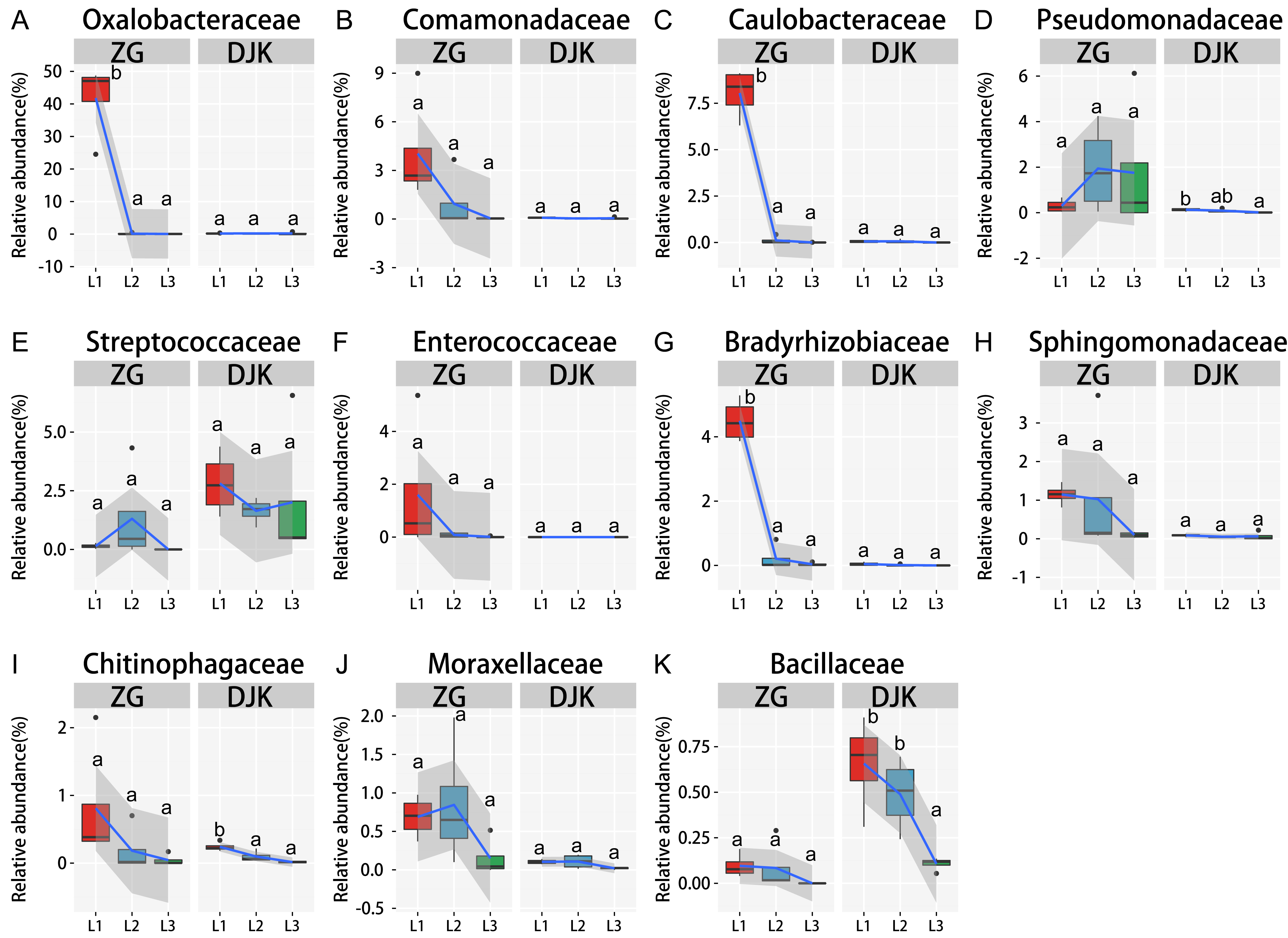

Supplement: FIGURE S4 — Relative abundance of bacterial families across the lifespan of larvae. (A–K) Boxplots showing the mean abundance of bacteria in the larval gut. In every panel, the left side represents the population collected from ZG (n = 4), and the right side represents the population collected from DJK (n = 4). Multiple comparisons were performed in the two group separately. Different letters indicate a significant difference between different instars in each group (p < 0.05, one-way ANOVA, Tukey post hoc test). The lines show median values per region window, and the shaded area denotes the estimated 95% confidence interval. [file Image_4.JPEG]

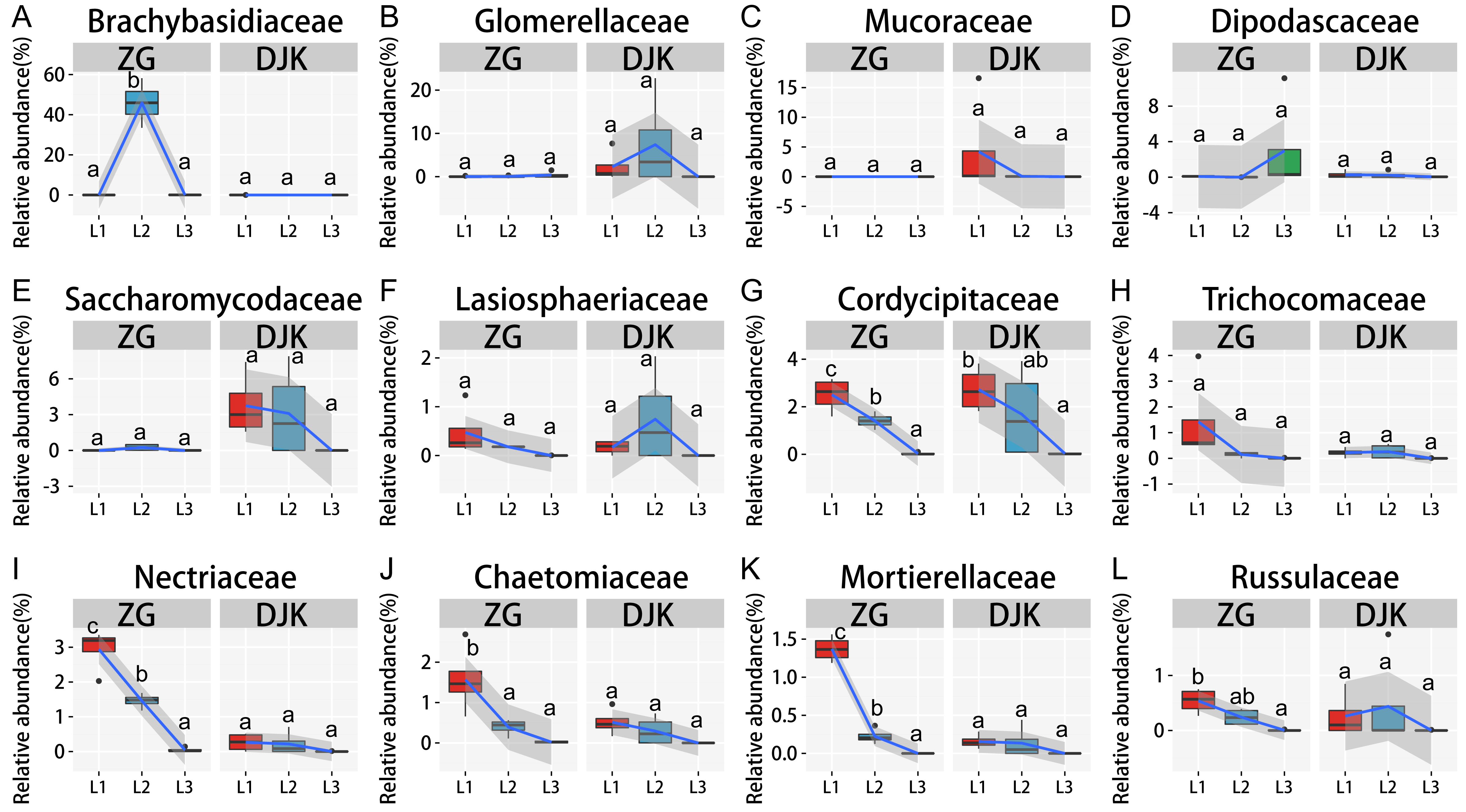

Supplement: FIGURE S5 — Relative abundance of fungi across the lifespan of larvae. (A–L) Boxplots showing the mean abundance of fungi in the larval gut. In every panel, the left side represents the population collected from ZG (n = 4), and the right side represents the population collected from DJK (n = 4). Multiple comparisons were performed in the two group separately. Different letters indicate a significant difference between different instars in each group (p < 0.05, one-way ANOVA, Tukey post hoc test). The lines show median values per region window, and the shaded area denotes the estimated 95% confidence interval. [file Image_5.JPEG]

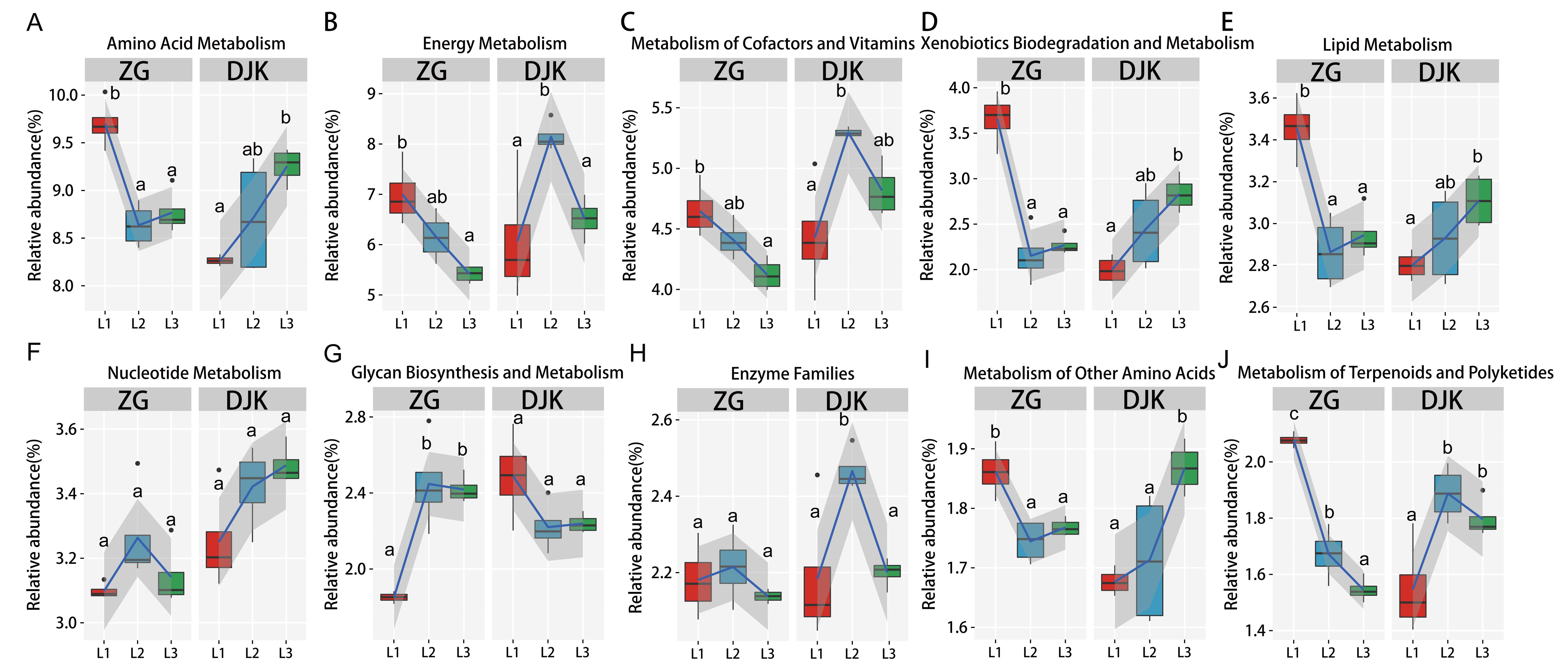

Supplement: FIGURE S6 — Predicted gut bacterial functions of larvae from two field populations. (A–J) the inferred metabolic pathways amino acid metabolism, energy metabolism, metabolism of cofactors and vitamins, xenobiotics biodegradation and metabolism, lipid metabolism, nucleotide metabolism, glycan biosynthesis and metabolism, enzyme families, metabolism of other amino acids and metabolism of terpenoids and polyketides are shown at the second hierarchical level (n = 4). Multiple comparisons were performed in the two group separately. Different letters indicate a significant difference between different instars in each group (p < 0.05, one-way ANOVA, Tukey post hoc test). [file Image_6.JPEG]
