# Supplementary material for: Similar Shift Patterns in Gut Bacterial and Fungal Communities Across the Life Stages of Bactrocera minax Larvae From Two Field Populations
Source: Front Microbiol. 2019 Oct 9;10:2262. doi: 10.3389/fmicb.2019.02262 (PMC6794421; doi:10.3389/fmicb.2019.02262)
Supplement: TABLE S1 — Comparisons of the relative abundance of gut bacteria from larval and adult samples at family level. [file Table_1.DOCX]

**Table S1**. Comparisons of the relative abundance of gut bacteria from larval and adult samples at family level.

| **Family** | **ZG-L1** | **ZG-L2** | **ZG-L3** | **DJK-L1** | **DJK-L2** | **DJK-L3** | **AMI** | **AFI** |
| --- | --- | --- | --- | --- | --- | --- | --- | --- |
| **Enterobacteriaceae** | 14.09$\pm$3.67^a^ | 87.20$\pm$6.14^b^ | 63.20$\pm$2.51^ab^ | 87.37$\pm$2.31^b^ | 54.71$\pm$23.72^ab^ | 15.17$\pm$2.46^a^ | 77.78$\pm$3.80^b^ | 79.26$\pm$6.43^b^ |
| **Acetobacteraceae** | 3.66$\pm$0.54^a^ | 0.44$\pm$0.28^a^ | 13.82$\pm$4.96^a^ | 0.17$\pm$0.06^a^ | 32.59$\pm$19.04^ab^ | 56.55$\pm$2.38^b^ | 1.39$\pm$1.29^a^ | 0.30$\pm$0.07^a^ |
| **Leuconostocaceae** | 0^a^ | 0.02$\pm$0.01^a^ | 2.16$\pm$1.08^a^ | 0.16$\pm$0.04^a^ | 2.57$\pm$1.43^ab^ | 6.63$\pm$1.48^b^ | 0.02$\pm$0.02^a^ | 0^a^ |
| **Lactobacillaceae** | 0.04$\pm$0.02^a^ | 0^a^ | 0.41$\pm$0.14^a^ | 0.12$\pm$0.07^a^ | 0.59$\pm$0.45^a^ | 2.07$\pm$0.38^b^ | 0^a^ | 0.05$\pm$0.05^a^ |
| **Xanthomonadaceae** | 0.19$\pm$0.05^a^ | 0.17$\pm$0.10^a^ | 16.26$\pm$3.23^b^ | 0.26$\pm$0.10^a^ | 2.94$\pm$1.73^a^ | 0.90$\pm$0.51^a^ | 0.33$\pm$0.16^a^ | 0.17$\pm$0.08^a^ |
| **Brucellaceae** | 6.28$\pm$4.45^a^ | 1.57$\pm$1.50^a^ | 0.19$\pm$0.16^a^ | 2.34$\pm$0.56^a^ | 1.18$\pm$0.50^a^ | 0.17$\pm$0.05^a^ | 0.02$\pm$0.02^a^ | 0.07$\pm$0.03^a^ |
| **Streptococcaceae** | 0.14$\pm$0.05^a^ | 1.31$\pm$1.02^a^ | 0^a^ | 2.81$\pm$0.67^a^ | 1.64$\pm$0.27^a^ | 2.01$\pm$1.52^a^ | 3.78$\pm$2.25^a^ | 2.46$\pm$0.15^a^ |
| **Moraxellaceae** | 0.69$\pm$0.13^a^ | 0.84$\pm$0.40^a^ | 0.15$\pm$0.12^a^ | 0.10$\pm$0.01^a^ | 0.11$\pm$0.05^a^ | 0.02$\pm$0.01^a^ | 4.20$\pm$1.45^b^ | 9.40$\pm$1.95^c^ |
| **unclassified_Bacteria** | 11.09$\pm$4.23^a^ | 3.47$\pm$2.68^a^ | 0.53$\pm$0.37^a^ | 1.67$\pm$0.17^a^ | 1.73$\pm$1.16^a^ | 14.38$\pm$7.02^a^ | 8.00$\pm$1.40^a^ | 3.75$\pm$1.58^a^ |

Abbreviations: DJK, Danjiangkou in the Hubei Province; ZG, Zigui in the Hubei Province; L1, first instar larvae; L2, second instar larvae; L3, third instar larvae; AMI, adult male-intestine; AFI, adult female-intestine.

Multiple comparisons were performed with one-way analysis of variance and Tukey’s test using SPSS 20. Abundance with the same letter are not significantly different (P>0.05). The bacterial 16S rRNA data was from Wang et al., 2014.
