# Supplementary material for: Similar Shift Patterns in Gut Bacterial and Fungal Communities Across the Life Stages of Bactrocera minax Larvae From Two Field Populations
Source: Front Microbiol. 2019 Oct 9;10:2262. doi: 10.3389/fmicb.2019.02262 (PMC6794421; doi:10.3389/fmicb.2019.02262)
Supplement: TABLE S2 — The nearest sequenced taxon index (NSTI) of larval samples. [file Table_2.DOCX]

**Table S2**. The nearest sequenced taxon index (NSTI) of larval samples.

| Samples | Metric | Value |
| --- | --- | --- |
| ZG-L1 | Weighted NSTI | 0.14$\pm$0.02 |
| ZG-L2 | Weighted NSTI | 0.11$\pm$0.01 |
| ZG-L3 | Weighted NSTI | 0.06$\pm$0.01 |
| DJK-L1 | Weighted NSTI | 0.11$\pm$0.03 |
| DJK-L2 | Weighted NSTI | 0.18$\pm$0.01 |
| DJK-L3 | Weighted NSTI | 0.09$\pm$0.01 |

NSTI represents the average phylogenetic distance between all microbial OTU in a sample and its nearest relative with a sequenced reference genome, the lower the value, the more accuracy of PICRUSt.
